# Sleep Quality and Mental Health of High-Level Esports Competitors: A Cross-Sectional Study

**DOI:** 10.3390/healthcare14050582

**Published:** 2026-02-26

**Authors:** Hiroaki Yamamoto, Hiroyuki Muraoka, Ken Inada

**Affiliations:** Department of Psychiatry, Kitasato University School of Medicine, Sagamihara 252-0374, Japan

**Keywords:** esports, sleep quality, psychological distress, depression, mental health

## Abstract

**Background:** Sleep quality and mental health are important concerns for esports competitors. However, epidemiological data regarding sleep quality and psychological distress among high-level esports players remain limited. **Objective:** This study aimed to examine sleep quality and mental health status among high-level esports competitors in Japan and to identify factors associated with psychological distress. **Methods:** This cross-sectional study included 275 competitors (269 males, 3 females, and 3 who did not report sex) participating in the 2023 National Prefectural Esports Championship. Sleep quality was assessed using the Pittsburgh Sleep Quality Index (PSQI), psychological distress using the Kessler Psychological Distress Scale (K6), and depressive symptoms using the Patient Health Questionnaire-9 (PHQ-9). Multivariable logistic regression analyses were performed to identify associated factors. **Results:** The response rate was 61.8% (275/445). Poor sleep quality (PSQI > cut-off) was observed in 38.5% of competitors. Based on the K6, 24.4% reported psychological distress above the mild threshold, and 29.5% reported mild or greater depressive symptoms according to the PHQ-9. Overall mental health levels were comparable to those reported in previous studies of general populations. Nighttime esports training was significantly associated with psychological distress (adjusted odds ratio 3.80; 95% confidence interval 1.50–9.64; *p* = 0.005). **Conclusions:** More than one-third of Japanese esports competitors experience poor sleep quality, and approximately one-quarter report mild or greater psychological distress. Nighttime esports training may be an important factor associated with mental health among competitors. Further longitudinal studies are warranted to clarify temporal relationships and to examine whether reducing nighttime training is associated with improved mental health outcomes among esports competitors.

## 1. Introduction

Esports (electronic sports) encompasses all forms of entertainment, competition, and sports conducted using electronic devices. Specifically, the term describes the interpretation of computer and video games as competitive sports. Esports is rapidly emerging as a new competitive domain and was featured for the first time as an official medal event at the 2023 Asian Games, alongside traditional sports, such as athletics and swimming. Additionally, athletes also engage in esports as a recreational sport. There is a wide range of competitors, from those who enjoy esports recreationally to those who pursue serious competitive pursuits. As of November 2024, 335 professional license holders in 18 game titles were recognized by the Japan esports Union (JeSU), the national governing body in Japan. As more players engage in competitive gaming, concerns about competitors’ sleep hygiene and mental health are steadily increasing [1].

Regarding the relationship between screen media device use incorporated into video games and sleep, device use at bedtime was significantly associated with insufficient sleep time, poor sleep quality, and excessive daytime sleepiness [2]. Esports players who use video screens for extended periods are at risk of sleep disorders, as many have a nocturnal lifestyle [3], and sleep disorders may reduce player performance [4]. Additionally, esports players have a higher prevalence of mental health symptoms than the general population [5]. Video game training time is strongly correlated with depressive symptoms (Center for Epidemiologic Studies Depression Scale, CES-D score) [3], suggesting that esports are not only associated with sleep disorders but also with depression, anxiety, apathy, excessive dependence, and gaming disorders [6].

It has been reported that the sleep health of esports players does not differ from that of their peers. However, many of these players are “night owls” [7], and sleep issues are more likely to be associated with Internet Gaming Disorder (IGD), as defined in the fifth edition of the Diagnostic and Statistical Manual of Mental Disorders (DSM-5), as well as Gaming Disorder (GD), as defined in the 11th edition of the International Classification of Diseases (ICD-11) [8]. These issues are also linked to the intensity of video game playing or the degree of immersion in gaming [9,10,11,12].

However, there are conflicting reports regarding the positive and negative impacts of esports on mental health [13]. Research examining the relationship between esports and sleep hygiene, anxiety, and depressive symptoms is still limited, hindering a comprehensive understanding of these effects.

Despite Japan’s thriving esports scene, insufficient information on sleep hygiene and mental health in high-level esports has been presented. To address this gap, we conducted a cross-sectional survey among high-level esports competitors who advanced through regional qualifiers and participated in the final stage of a national-scale tournament in Japan.

Based on previous findings, we hypothesized that poor sleep quality and psychological distress would be prevalent among high-level esports competitors, and that nighttime esports training would be associated with psychological distress independently of total esports training duration.

## 2. Methods

### 2.1. Study Design and Participants

This cross-sectional study used data from a voluntary questionnaire. The target population comprised 445 players who had advanced through the regional qualifiers of the 2023 National esports Championship. This annual competition involves players from various regions of Japan, with those who pass the qualifying rounds representing their respective prefectures to compete in the championship at the host region. The first round of the competition was held online for each game title, and according to the organizers, approximately 70,000 people participated [14].

The main competition was held at KAGOSHIMA on 25 and 26 November 2023. The competition featured six game titles and two age categories. The titles included Identity V (for players over 12 years old), eFootball (open category), Gran Turismo 7 (for both under 18 years old/over 18 years old), Shadowverse (junior high school, high school, college students), Puzzle and Dragons (open category), and Puyo Puyo eSports (elementary school students/open category).

The purpose of the study, procedure, and voluntary nature of the survey were explained to the participants, emphasizing that there would be no disadvantages for those who chose not to participate. Their consent was obtained before the survey was conducted. The survey instrument was created using Google Forms (Google Incorporated, 1600 Amphitheatre Parkway, Mountain View, CA, USA), and the participants accessed and completed the survey instrument via the provided survey URL. All players were given the opportunity to complete the survey in a private and confidential manner. Participation in the questionnaire was voluntary and limited to competitors who attended the final event, and therefore the sample does not represent all eligible competitors who participated in the regional qualifiers.

### 2.2. Survey Items

Survey items included sex, age, occupation/school attendance status, esports professional license status, specialized game category, total physical training time per week, total esports training time per week, and esports training time slots (8–20:daytime/20–8:nighttime).

Validated screening tools were used to evaluate sleep hygiene and mental health. The Pittsburgh Sleep Quality Index (PSQI) was used to assess sleep quality [15]. The PSQI is a 19-item self-reported measure with scores for seven groups: subjective sleep quality, sleep latency, sleep duration, sleep efficiency, sleep disturbances, daytime dysfunction, and sleep medication use. The score range is 0–21 points, with a total score ≥5.5 indicating the presence of a sleep disorder [15,16].

The Japanese version of the Kessler-6 (K6) was used to measure anxiety and depressive symptoms. This self-report tool assesses mental distress, such as irritability, fatigue, hopelessness, and agitation, over the past 30 days, using a 5-point scale for each of the six items. Scores range from 0 to 24, with higher scores indicating more severe symptoms [17,18,19]. The K6 Japanese version was used in this study [19,20,21].

Studies examining appropriate cut-off points for the Japanese version of the K6 have categorized levels of mental distress based on scores as follows: normal (0–4), mild (5–10), moderate (10–12), and severe (13–24) [19,21]. This classification was adopted for the K6 in this study.

Furthermore, the Japanese version of the nine-item Patient Health Questionnaire-9 (PHQ-9) was used to measure depressive symptoms [22,23,24,25,26]. Scores range from 0 to 27, with higher scores indicating more severe depressive symptoms. The cut-off points for the PHQ-9 are as follows: normal (≤4), mild or worse (5–9), and moderate or worse (≥10) [24,25]. The PHQ-9 is a validated tool for investigating depressive symptoms in student athletes [26].

### 2.3. Statistical Analysis

Statistical analyses were performed using the JMP Pro version 17 software for Windows (SAS Institute Inc., Cary, NC, USA). Descriptive statistics were used to summarize data. For continuous variables, means, standard deviations, medians, and interquartile ranges were calculated. Student’s *t*-test was used for normally distributed continuous variables, while Welch’s *t*-test was applied for those with unequal distributions. Fisher’s exact test (two-tailed) was used to assess the relationships between categorical data in contingency tables. The Kruskal–Wallis non-parametric test was employed to compare continuous variables across groups, and the Wilcoxon test, a non-parametric method, was used for the pairwise comparisons of continuous variables among categories with three or more groups.

The significance level was set at *p* < 0.05 for comparisons between two groups. For comparisons involving three or more groups, the *p*-value was adjusted using Bonferroni’s *p*-value based on the number of combinations. Specifically, a significance level of *p* < 0.017 was used for sex and esports training time slots (3 combinations), *p* < 0.0033 for age and occupation (15 combinations), and *p* < 0.0018 for game categories (28 combinations).

Multivariate logistic regression analyses were conducted for the PSQI, K6, and PHQ-9 to analyze the factors associated with each above-threshold condition. The objective variable was defined as exceeding the threshold for each indicator, with explanatory variables including characteristics (sex, age, occupation/school attendance status) and training habits (physical training time [h/week], esports training time [h/week], and esports training time slots [8–20, 20–8]). Age was included as a covariate in all multivariable logistic regression models to account for the wide age range of participants. The number of explanatory variables was limited to more than 10 observations per variable [27]. Only significant equations (*p* < 0.05) in the overall model evaluation and non-significant variables (*p* > 0.05) in the test of lack of fit were included.

## 3. Results

### 3.1. Participant Sampling

A total of 445 individuals were initially enrolled in this study. After excluding individuals who declined to participate or whose data were incomplete, 275 (61.8%) participants were included (269 males, 3 females, and 3 participants whose sex was not specified).

### 3.2. Participant Characteristics

Table 1 presents the characteristics of the participants. The sex distribution was 97.8% male, 1.1% female, and 1.1% non-responsive. The mean age of participants was 24.8 ± 11.8 years. The occupation/school attendance status was as follows: working adults (42.9%), followed by high school students (22.2%), college/junior college/vocational school students (18.9%), junior high school students (12.7%), elementary school students (1.8%), and others (1.5%). Racing (67.6%) was the most common specialized game category. When asked when they usually trained in esports, 71.6% reported training at night (8 PM to 8 AM), 24.3% during the day (8 AM to 8 PM), and 4.0% did not provide training time information.

### 3.3. Relationship of Sleep Dysfunctions with Demographic Characteristics

Table 2 shows the relationship between sleep dysfunction and the demographic characteristics. In the study population, 38.5% reported a sleep disorder exceeding the screening threshold (≥5.5). The overall mean PSQI score was 5.3 ± 3.1.

Prevalence of sleep dysfunction did not differ significantly by sex, professional license status, or gaming category. However, significant differences were observed in PSQI scores by occupation and schooling status, with junior high school students scoring significantly lower than college students and adults.

The overall mean sleep duration was 6.5 ± 1.2 h. There were significant differences in sleep duration across age groups, with those aged 14 years and younger sleeping significantly longer than those aged 15–19, 25–29, 30–34, and ≥35 years. Sleep duration differed significantly by occupation and schooling status, with primary school children sleeping significantly longer than high school students and adults, and secondary school students sleeping significantly longer than high school students and adults.

Physical training time (hours/week) was significantly longer in the group without sleep disorders (<5.5) than in the group with sleep disorders (≥5.5). However, no significant differences were observed in esports training time (hours/week) by sleep disorder status.

The mean PSQI score and sleep duration differed significantly between esports training time slots: the group practicing from 8 AM to 8 PM (daytime) had substantially lower sleep disturbance scores and longer sleep duration than those practicing 8 PM to 8 AM (nighttime) and the non-responding group.

### 3.4. Relationship Between Anxiety and Depression Symptoms and Demographic Characteristics (K6)

Table 3 illustrates the relationship between anxiety and depression symptoms and demographic characteristics. No significant differences in mean K6 scores were observed by sex, age group, occupation, schooling status, or professional license status. The overall mean K6 score was 2.9 ± 4.3. Most participants (75.6%) scored within the normal range (<5), while 24.4% exhibited mild symptoms (≥5), and 4.4% reported severe symptoms (≥13). The length of time spent in physical and esports training did not differ significantly by symptom status.

There were significant differences in the mean K6 scores and number of individuals exceeding the threshold for mild symptoms by esports training time slots. Notably, the mean K6 scores differed significantly by esports training time slots, with participants training at night (8 PM–8 AM) reporting higher scores than those training during the day (8 AM–8 PM).

### 3.5. Relationship of Depression Symptoms with Demographic Characteristics (PHQ-9)

Table 4 shows the relationships between depressive symptoms and demographic characteristics. The overall mean PHQ-9 score was 3.9 ± 4.9. Among the total population, 70.6% scored within the normal range (<5), 29.5% reported more than mild depressive symptoms (≥5), and 12.4% had severe symptoms (≥10). No significant differences were found based on sex, or professional license status. However, significant differences in PHQ-9 scores were observed by age group, occupation and schooling status, with 20–24 year olds scoring higher than those aged 14 or younger, and college students scoring significantly higher scores than secondary school students and adults. Participants without depressive symptoms engaged in more hours of physical training per week than those with depressive symptoms, while hours spent on esports training did not differ significantly.

The PHQ-9 scores showed significant differences based on esports training time slots, with the group practicing during nighttime having significantly higher scores than the group practicing during daytime.

### 3.6. Multivariate Logistic Regression Analysis of Factors Related to K6 Scores ≥ 5

Table 5 presents the multivariate logistic regression analysis of factors associated with K6 scores ≥5. While the model did not predict the prevalence of symptoms based on PSQI or PHQ-9 scores, nighttime esports training was significantly associated with higher odds of anxiety and depression symptoms.

Participants training at night (8 PM–8 AM) had a 3.8-fold increased likelihood of reporting mild or greater symptoms than those training during the day (adjusted odds ratio, 3.80; 95% confidence interval, 1.50–9.64; *p* = 0.005).

## 4. Discussion

### 4.1. Key Results

This study aimed to understand sleep quality and mental health among high-level Japanese esports competitors. Three key findings emerged from the analysis.

First, regarding sleep hygiene, 38.5% of the competitors experienced sleep disturbances, as indicated by scores above the PSQI threshold.

Second, the prevalence of mental health concerns was notable, with 24.4% of competitors reporting anxiety and depressive symptoms above the mild threshold on the K6 index, while 29.5% exhibited depressive symptoms above the mild threshold on the PHQ-9 index.

Third, esports training at night (after 8 p.m.) was associated with increased likelihood of reporting mild or greater anxiety and depressive symptoms than esports training during the day.

### 4.2. Sleep Quality

A total of 38.5% of the athletes in the study experienced above-threshold sleep disturbances, with an overall mean PSQI score of 5.3 ± 3.1.

As shown in Supplementary Table S1, previous research in the general Japanese population reported a mean PSQI score of 5.7 ± 2.5 and an average sleep duration of 6.3 ± 1.1 among male high school students [28]. Similarly, high school male esports players in this study had a PSQI score of 5.2 ± 3.1 and sleep duration of 6.2 ± 0.8, with no significant differences observed between them and the general population. Likewise, male college students in the general Japanese population had a reported PSQI score of 4.8 ± 4.5 and an average sleep duration of 5.8 ± 1.4 h [29]. In comparison, male college esports players in this study had a PSQI score of 5.8 ± 3.3, which was not significantly different from that of the general population. However, the esports players reported a longer average sleep duration of 6.6 ± 1.4 h. In contrast, the mean PSQI score in the 20–29-year-old male group in the present study was significantly higher than that of the general population (5.8 ± 3.1 vs. 4.5 ± 2.1) [30].

Although excessive video game play and prolonged playing times have been reported to associated with sleep disturbances [6,10,11,12,31,32], the high school and college students in this study may have avoided “excessive” and “prolonged” playing durations highlighted in previous reports. Sleep deprivation has been reported to reduce esports performance [4]. Therefore, most of the high performers in this study successfully avoided sleep deprivation to enhance their competitive performance. However, for individuals in the 20–29-year age group, which is primarily composed of working adults, the challenge of balancing work with esports may negatively impact sleep hygiene.

Participants training at night demonstrated significantly higher PSQI scores and shorter sleep durations than those training during the day. This is consistent with previous reports that playing at night has been associated with sleep disturbances [2,10,11,12]. For instance, an experimental study examining the relationship between screen device usage and sleep in students aged 12–19 years reported that sleep hygiene improved when device use was limited after 21:00 [33]. Furthermore, in the present study, the symptomatic group with sleep disorders (PSQI ≥5.5) spent significantly less time on physical training than the symptom-free group, while the time spent on esports training did not differ significantly. Moderate physical exercise positively affects sleep [34,35]. Although the content and intensity of exercise were not investigated in this study, the potential benefits of exercise on sleep hygiene in esports competitors are worth investigating.

### 4.3. Prevalence of Anxiety and Depression

The prevalence of mental health concerns was notable, with 24.4% of competitors reporting anxiety and depressive symptoms above the mild threshold on the K6 index (with 4.4% showing severe symptoms), while 29.5% exhibited depressive symptoms above the mild threshold on the PHQ-9 index (with 12.4% showing severe symptoms).

In prior studies from Japan (Supplementary Table S1), K6 scores of 5.4 ± 4.5 were reported for general male college students (19 ± 1.0 years) [36] and 5.2 ± 4.6 for male college physical education students [37]. The K6 score of 3.4 ± 4.1 for the male college students in the present study was significantly lower than those reported in previous studies. In contrast, the PHQ-9 score of 5.4 ± 4.6 did not differ significantly from existing reports, including the mean score of 6.5 ± 5.2 for general male college students in Japan (20.8 ± 1.8 years) [38]. Similarly, no significant differences were observed in the incidence of K6 score ≥5 between the general male population (20–29 years) [39] and their peers in our study (24.7% vs. 28.4%).

Although the prevalence of anxiety and depression symptoms in esports players has been reported to be higher than that in the general population in previous studies [5,32], this did not apply to the present study. Conversely, esports players exhibited more favorable K6 scores, indicating fewer symptoms than those in the general group of college students.

While previous studies indicate conflicting findings regarding the positive and negative impacts of esports on mental health [13], the current study of high performers found either no effect or a positive impact on college students.

In contrast to previous studies [3], esports training duration did not significantly correlate with the K6 or PHQ-9 thresholds in the present study. However, earlier studies were based on a more restricted population, including specific game types (first-person shooter) and a relatively small number of professional players [17], which likely contributed to the higher likelihood of detecting a significant effect of esports training duration.

### 4.4. Relationship of Nighttime Training Habits with Anxiety and Depressive Symptoms

We found that esports training at night (after 8 p.m.) was associated with increased likelihood of reporting mild or greater anxiety and depressive symptoms than training during the day, suggesting a possible association with circadian rhythm disruption, although causality cannot be inferred from the present cross-sectional design.

Circadian rhythms are linked to the sleep–wake cycle, and disruption of circadian rhythms can lead to sleep disturbances [40]. Exposure to blue light emitted from electronic screens in the evening has been shown to suppress melatonin secretion and disrupt circadian rhythms, which may partly explain the observed association. It is also widely known that circadian rhythm disturbances increase the risk of developing mental health problems, including depressive symptoms [41,42,43].

Notably, the present study suggests that the timing of esports training may be relevant to these outcomes, independent of training duration. Unlike traditional sports, esports can be played at night, which is often necessary for individuals who work or attend school during the day. Maintaining physical fitness is key to both enjoying and excelling in esports. The classification of esports training time into daytime (08:00–20:00) and nighttime (20:00–08:00) was based on a pragmatic distinction between socially normative activity periods and biologically relevant nighttime hours commonly used in sleep research. However, this dichotomization may obscure more nuanced training patterns and does not account for individual chronotype differences. The relationship between nighttime training and mental health, including the possibility that shifting training away from nighttime hours may be associated with better mental health, should be examined in future longitudinal or interventional studies.

From a public health perspective, identifying modifiable lifestyle factors such as nighttime esports training may contribute to the development of preventive strategies for mental health among esports competitors.

### 4.5. Reverse Causality

Because of the cross-sectional design, the temporal direction of the observed association cannot be determined. It is possible that nighttime esports training is associated with psychological distress; however, individuals with pre-existing psychological distress may also preferentially engage in nighttime training. Therefore, the present findings should be interpreted strictly as associations.

### 4.6. Limitations

This study has several limitations. First, the cross-sectional design precludes any inference of causal relationships among the variables examined. Second, the data were collected using retrospective self-reports and may therefore be subject to recall and reporting biases related to participants’ interpretation of questions, response sincerity, and memory capacity.

Third, several potentially important confounders, including chronotype, caffeine consumption, screen exposure intensity and duration, prior mental health conditions, and sleep medication use, were not measured and could not be adjusted for. Residual confounding therefore cannot be ruled out, and the findings should be interpreted as exploratory associations. In addition, the moderate response rate and the inability to compare respondents with non-respondents introduce potential selection bias, limiting the interpretability of prevalence estimates.

Finally, the study population was predominantly male, drawn from a single country, and largely composed of competitors in one game category (racing games). As such, the findings cannot be generalized to esports players overall, particularly female competitors or those in other esports genres. This study is reported in accordance with the STROBE guidelines for cross-sectional studies [44].

## 5. Conclusions

Over one-third of Japanese esports competitors reported poor sleep quality, and approximately one-quarter reported mild or greater psychological distress. Nighttime esports training was associated with mental health among high-level esports competitors. Further longitudinal research is warranted to clarify temporal relationships and to examine whether shifting training away from nighttime hours is associated with better mental health. Given that a substantial proportion of competitors reported poor sleep quality, interventions to promote better sleep hygiene may be beneficial for overall health and potentially for performance. Specific strategies may include limiting nighttime training and screen exposure and encouraging regular sleep schedules, particularly for those in the 20–29-year age group who balance work and esports; however, the effectiveness of such strategies should be evaluated in future interventional or longitudinal studies.

## Figures and Tables

**Table 1:** Characteristics of esports athletes (n = 275).

Characteristic	Overall (n = 275)
**Sex**	
Male, n (%)	269 (97.8)
Female, n (%)	3 (1.1)
Did not report, n (%)	3 (1.1)
**Age (years)**	
Mean (SD)	24.8 (11.8)
Median (IQR)	20 (17–30)
**Distribution, n (%)**	
≤14	33 (12.0)
15–19	94 (34.2)
20–24	50 (18.2)
25–29	26 (9.5)
30–34	21 (7.6)
≥35	51 (18.5)
**Occupation/School attendance status, n (%)**	
Elementary school students	5 (1.8)
Junior high school students	35 (12.7)
High school students	61 (22.2)
College/junior college/vocational school students	52 (18.9)
Working adults	118 (42.9)
Others	4 (1.5)
**Professional license, n (%)**	
Held	16 (5.8)
Did not hold	259 (94.2)
**Specialized game category, n (%)**	
Fighting	1 (0.4)
Music/rhythm games	5 (1.8)
Racing	186 (67.6)
Puzzles	40 (14.5)
Sports	13 (4.7)
FPS/TPS	17 (6.2)
Digital card games	9 (3.3)
Other	4 (1.5)
**Physical training (hour/week)**	
Mean (SD)	5.7 (6.2)
Median (IQR) [minimum–maximum]	3.5 (1.4–7) [0–49]
**Esports training (hour/week)**	
Mean (SD)	17.5 (11.4)
Median (IQR) [minimum–maximum]	15 (10–21) [0–56]
**Esports training time slots**	
8 AM–8 PM (daytime)	67 (24.3)
8 PM–8 AM (nighttime)	197 (71.6)
No answer	11 (4.0)

Abbreviations: IQR, interquartile range; SD, standard deviation; FPS, first-person shooter; TPS, third-person shooter.

**Table 2:** Relationship between sleep dysfunctions and demographic characteristics.

Factor			PSQI Score					
	Mean (SD)	Median (IQR)	*p* Value	<5.5 n (%)	≥5.5 n (%)	*p* Value	Sleeping Time Mean (SD)	*p* Value
Overall	5.3 (3.1)	5 (3–7)		169 (61.5)	106 (38.5)		6.5 (1.2)	
**Sex**								
Male	5.3 (1.9)	5 (3–7)	0.92	165 (60)	104 (37.8)	1.00	6.5 (1.2)	0.72
Female	6.0 (5.2)	3 (3–12)		2 (0.7)	1 (0.4)		6.0 (1.0)	
Did not report	6.0 (3.6)	5 (3–10)		2 (0.7)	1 (0.4)		6.0 (1.7)	
**Age (years)**								
≤14	3.5 (2.7)	3 (1–5)	0.0052	27 (9.8)	6 (2.2)	0.14	7.5 (1.1)	<0.0001 *
15–19	5.3 (3.2)	4.5 (3–7)		56 (20.4)	38 (13.8)		6.3 (1.1)	
20–24	5.8 (3.1)	5 (3.8–8)		27 (9.8)	23 (8.4)		6.7 (1.4)	
25–29	5.8 (3.1)	5 (4–7)		16 (5.8)	10 (3.6)		6.5 (0.8)	
30–34	6.0 (2.8)	5 (4–7)		11 (4.0)	10 (3.6)		6.0 (1.3)	
≥35	5.4 (2.6)	5 (3–7)		32 (11.6)	19 (6.9)		6.2 (1.1)	
**Occupation/School attendance status**								
Elementary school students	1.8 (2.0)	2 (0–3.5)	0.0002 †	5 (1.8)	0 (0.0)	0.02	8.4 (0.5)	<0.0001 ‡
Junior high school students	3.7 (2.6)	3 (1–5)		28 (10.2)	7 (2.6)		7.3 (1.1)	
High school students	5.2 (3.1)	4 (3–7)		37 (13.5)	24 (8.7)		6.2 (0.8)	
College students (including junior college and vocational school students)	5.9 (3.4)	5 (3–7.8)		27 (9.8)	25 (9.1)		6.5 (1.4)	
Working adults	5.6 (2.9)	5 (4–7)		71 (25.8)	47 (17.1)		6.2 (1.1)	
Others	6.8 (1.7)	6.5 (5.3–8.5)		1 (0.4)	3 (1.1)		7.3 (1.5)	
**Professional license**								
Held	4.4 (2.5)	4 (3–5.8)	0.24	12 (4.4)	4 (1.5)	0.30	6.8 (1.7)	0.35
Did not hold	5.3 (3.1)	5 (3–7)		157 (57.1)	102 (37.1)		6.4 (1.2)	
Physical training (hour/week)				6.1 (6.6)	5.1 (5.5)	0.007		
Esports training (hour/week)				16.1 (11.0)	19.8 (11.8)	0.33		
**Esports training time slots**								
8 AM–8 PM (daytime)	4.0 (2.4)	4 (2–6)	0.0007 §	49 (17.8)	18 (6.6)	0.074	6.9 (1.2)	0.0002 ||
8 PM–8 AM (nighttime)	5.6 (3.1)	5 (3–7)		114 (41.5)	83 (30.2)		6.4 (1.2)	
No answer	6.5 (3.2)	5 (4–10)		6 (2.2)	5 (1.8)		5.6 (1.4)	

Abbreviations: IQR, Interquartile Range; SD, standard deviation. * *p* < 0.0001 between age groups ≤14 and 15–19, 30–34, and ≥35. † *p* = 0.0001 between age groups ≤14 and 25–29. ‡ *p* < 0.0001 between junior high school students and high school students/working adults. *p* = 0.0001 between elementary school students and high school students. *p* = 0.0003 between elementary school students and working adults. § *p* = 0.0004 between 8 AM and 8 PM (daytime) and 8 PM–8 AM (nighttime). *p* = 0.016 between 8 AM and 8 PM (daytime) and no answer. || *p* = 0.0003 between 8 AM and 8 PM (daytime) and 8 PM–8 AM (nighttime). *p* = 0.005 between 8 AM and 8 PM (daytime) and no answer.

**Table 3:** Relationship between anxiety and depression symptoms and demographic characteristics.

Factor		K6 Score							
	Mean (SD)	Median (IQR)	*p* Value	<5 n (%)	≥5 n (%)	*p* Value	<13 n (%)	≥13 n (%)	*p* Value
Overall	2.9 (4.3)	1(0–4)		208 (75.6)	67 (24.4)		263 (95.6)	12 (4.4)	
**Sex**									
Male	2.9 (4.3)	1 (0–4)	0.93	204 (74.2)	65 (23.6)	0.45	257 (93.5)	12 (4.4)	1.00
Female	3.3 (5.8)	0 (0–10)		2 (0.7)	1 (0.4)		3 (1.1)	0 (0.0)	
Did not report	3.0 (4.4)	1 (0–8)		2 (0.7)	1 (0.4)		3 (1.1)	0 (0.0)	
**Age (year)**									
≤14	1.7 (2.8)	0 (0–3.5)	0.14	29 (10.6)	4 (1.5)	0.32	33 (12.0)	0 (0.0)	0.55
15–19	2.7 (4.3)	1 (0–4)		72 (26.2)	22 (8.0)		91 (33.1)	3 (1.1)	
20–24	3.8 (4.6)	2 (0–6)		33 (12.0)	17 (6.2)		46 (16.7)	4 (1.5)	
25–29	3.0 (3.9)	2 (0–3.25)		21 (7.6)	5 (1.8)		25 (9.1)	1 (0.4)	
30–34	3.4 (4.8)	2 (0–5)		16 (5.8)	5 (1.8)		20 (7.3)	1 (0.4)	
≥35	2.9 (4.7)	1 (0–5)		37 (13.5)	14 (5.1)		48 (17.5)	3 (1.1)	
**Occupation/School attendance status**									
Elementary school students	1.2 (2.7)	0 (0–3)	0.16	4 (1.5)	1 (0.4)	0.19	5 (1.8)	0 (0.0)	0.032
Junior high school students	1.8 (2.7)	0 (0–4)		31 (11.3)	4 (1.5)		35 (12.7)	0 (0.0)	
High school students	2.7 (4.4)	1 (0–4)		47 (17.1)	14 (5.1)		58 (21.1)	3 (1.1)	
College students (including junior college and vocational school students)	3.5 (4.1)	1 (0–6.75)		35 (12.7)	17 (6.2)		51 (18.6)	1 (0.4)	
Working adults	3.0 (4.5)	1 (0–4.25)		89 (32.4)	29 (10.6)		112 (40.7)	6 (2.2)	
Others	7.5 (7.5)	8 (0.5–14)		2 (0.7)	2 (0.7)		2 (0.7)	2 (0.7)	
**Professional license**									
Held	1.9 (2.6)	1 (0–2.75)	0.84	14 (5.1)	2 (0.7)	0.37	16 (5.8)	0 (0.00)	1.00
Did not hold	2.9 (4.4)	1 (0–5)		194 (70.6)	65 (23.6)		247 (89.8)	12 (4.4)	
Physical training (hour/week)				6.1 (6.5)	4.6 (5.0)	0.07	5.8 (6.3)	4.7 (4.3)	0.75
Esports training (hour/week)				17.1 (11.4)	18.8 (11.4)	0.28	17.5 (11.6)	18.3 (8.2)	0.50
**Esports training time slots**									
8 AM–8 PM (daytime)	1.3 (2.3)	0 (0–2)	0.001 *	61 (22.2)	6 (2.2)	0.0009	67 (24.4)	0 (0.0)	0.11
8 PM–8 AM (nighttime)	3.5 (4.6)	2 (0–6)		138 (50.2)	59 (21.5)		186 (67.6)	11 (4.0)	
No answer	2.1 (4.8)	0 (0–1)		9 (3.3)	2 (0.7)		10 (3.6)	1 (0.4)	

Abbreviations: IQR, interquartile range; SD, standard deviation. * *p* = 0.0005 between 8 AM and 8 PM (daytime) and 8 PM–8 AM (nighttime).

**Table 4:** Relationship between depression symptoms and demographic characteristics.

Factor		PHQ-9 Score							
	Mean (SD)	Median (IQR)	*p* Value	<5 n (%)	≥5 n (%)	*p* Value	<10 n (%)	≥10 n (%)	*p* Value
Overall	3.9 (4.9)	2 (0–6)		194 (70.6)	81 (29.5)		241 (87.6)	34 (12.4)	
**Sex**									
Male	3.9 (4.8)	2 (0–6)	0.69	189 (68.7)	80 (29.1)	0.80	236 (85.8)	33 (12.0)	0.55
Female	5.0 (8.7)	0 (0–15)		2 (0.7)	1 (0.4)		2 (0.7)	1 (0.4)	
Did not report	1.7 (1.5)	2 (0–3)		3 (1.1)	0 (0.0)		3 (1.1)	0 (0.0)	
**Age (year)**									
≤14	2.0 (2.9)	1 (0–3)	0.01 *	30 (10.9)	3 (1.1)	0.01	31 (11.3)	2 (0.7)	0.36
15–19	4.1 (4.8)	3 (0–6)		66 (24.0)	28 (10.2)		79 (28.7)	15(5.5)	
20–24	5.6 (5.6)	4 (1.75–8.25)		28 (10.2)	22 (8.0)		41 (14.9)	9 (3.3)	
25–29	3.1 (3.3)	2 (1–4.25)		20 (7.3)	6 (2.2)		25 (9.1)	1 (0.4)	
30–34	4.4 (5.2)	3 (0–8)		12 (4.4)	9 (3.3)		19 (6.9)	2 (0.7)	
≥35	3.3 (5.2)	1(0–6)		38 (13.8)	13 (4.7)		46 (16.7)	5 (1.8)	
**Occupation/School attendance status**									
Elementary school students	0.6 (0.9)	0 (0–1.5)	0.002 †	5 (1.8)	0 (0.0)	0.01	5 (1.8)	0 (0.0)	0.02
Junior high school students	2.3 (2.9)	2 (0–3)		30 (10.9)	5 (1.8)		33 (12.0)	2 (0.7)	
High school students	4.1 (5.1)	2 (0–6)		44 (16.0)	17 (6.2)		51 (18.6)	10 (3.6)	
College students (including junior college and vocational school students)	5.6 (4.8)	4 (2–9)		28 (10.2)	24 (8.7)		41 (14.9)	11 (4.0)	
Working adults	3.6 (4.8)	2 (0–6)		85 (30.9)	33 (12.0)		109 (39.6)	9 (3.3)	
Others	8.8 (10.4)	6 (0.5–19.75)		2 (0.7)	2 (0.7)		2 (0.7)	2 (0.7)	
**Professional license**									
Held	3.6 (4.5)	2 (0.25–4.5)	0.78	12 (4.4)	4 (1.5)	0.78	14 (5.1)	2 (0.7)	1.00
Did not hold	3.9 (4.9)	2 (0–6)		182 (66.2)	77 (28.0)		227 (82.6)	32 (11.6)	
Physical training (hour/week)				6.4 (6.7)	4.3 (4.6)	0.01	6.0 (6.3)	4.2 (5.4)	0.07
Esports training (hour/week)				17.1 (10.9)	18.5 (12.6)	0.52	17.1 (11.2)	20.1 (12.6)	0.18
**Esports training time slots**									
8 AM–8 PM (daytime)	2.3 (3.2)	1 (0–3)	0.001 ‡	55 (20.0)	12 (4.4)	0.01	64 (23.3)	3 (1.1)	0.047
8 PM–8 AM (nighttime)	4.5 (5.1)	3 (1–7)		129 (46.9)	68 (24.7)		167 (60.7)	30 (10.9)	
No answer	3.1 (7.0)	1 (0–3)		10 (3.6)	1 (0.4)		10 (3.6)	1 (0.4)	

Abbreviations: IQR, interquartile range; SD, standard deviation. * *p* = 0.0005 between age groups <=14 and 20–24. † *p* = 0.0005 between junior high school students and college students. *p* = 0.0013 between college students and working adults. ‡ *p* = 0.0008 between 8 AM and 8 PM (daytime) and 8 PM–8 AM (nighttime).

**Table 5:** Multivariate logistic regression analysis of K6 score ≥5-related factors (n = 275).

			Multivariate Logistic Regression Analysis		
	K6 Score < 5	K6 Score ≥ 5	Adjusted OR	95% CI	*p* Value
Sex					
Male	204	65	reference	reference	reference
Female	2	1	1.17	0.10–13.89	0.90
Did not report	2	1	1.74	0.14–21.28	0.67
Age (years)					
<20	101	26	reference	reference	reference
≥20	107	41	1.35	0.46–3.98	0.58
Occupation/School attendance status					
Elementary school students	4	1	reference	reference	reference
Junior high school students	31	4	0.45	0.04–5.69	0.54
High school students	47	14	0.79	0.07–8.41	0.84
College students (including junior college and vocational school students)	35	17	0.86	0.07–10.45	0.91
Working adults	89	29	0.53	0.04–6.83	0.63
Others	2	2	1.71	0.07–44.80	0.75
Physical training (hour/week), mean [SD], (per 1)	6.1 [6.5]	4.6 [5.0]	0.96	0.91–1.01	0.14
Esports training (hour/week), mean [SD], (per 1)	17.1 [11.4]	18.8 [11.4]	1.01	0.98–1.04	0.40
Esports training time slots					
8 AM–8 PM (daytime)	61	6	reference	reference	reference
8 PM–8 AM (nighttime)	138	59	**3.80**	**1.50–9.64**	0.005 *
no answer	9	2	2.16	0.36–13.10	0.40

Reported as n unless otherwise specified. Abbreviations: SD, standard deviation; OR, odds ratio; CI confidence interval. A multivariate logistic regression analysis was used after adjustment for sex, age, occupation/school attendance status, physical training (hour/week), esports training (hour/week), and esports training time slots. * Data with *p*-value < 0.05 and 95% confidence interval not crossing 1 are shown in bold. Overall model evaluation, *p* = 0.049; Lack of Fit test, *p* = 0.24; observation 275.

## Data Availability

The data presented in this study are available from the corresponding author upon reasonable request. Due to ethical restrictions and the protection of participant anonymity, the data are not publicly available.

## Supplementary Materials

The following supporting information can be downloaded at: https://www.mdpi.com/article/10.3390/healthcare14050582/s1, Table S1: Comparison of the results of this study with previous studies.

## Abbreviations

Esports	electronic sports
K6	Kessler-6
PHQ-9	Patient Health Questionnaire-9
PSQI	Pittsburgh Sleep Quality Index
