# Supplementary material for: Sleep Quality and Mental Health of High-Level Esports Competitors: A Cross-Sectional Study
Source: Healthcare (Basel). 2026 Feb 26;14(5):582. doi: 10.3390/healthcare14050582 (PMC12984269; doi:10.3390/healthcare14050582)
Supplement: Supplementary file 1 [file healthcare-14-00582-s001.zip › healthcare-4150421-supplementary.pdf]

Supplementary Table S1. Comparison of the results of this study with previous studies

Previous studies					Data covered in this study		
**Authors**	**Subject populations**	**n**	**Type of data**	**Content of data**	**n**	**mean (SD)**	**P Value**
Tagaya H, Uchiyama M, Ohida T, et al. [28]	General Japanese high school students (male)	3478	PSQI score (mean, SD)	5.7 (2.5)	61	5.2 (3.1)	0.22
			Sleeping time (hours/day) (mean, SD)	6.3 (1.1)	61	6.2 (0.8)	0.34
Horiuchi M, Oda S. [29]	General Japanese college students (male)	80	PSQI score (mean, SD)	4.8 (4.5)	51	5.8 (3.3)	0.15
			Sleeping time (hours/day) (mean, SD)	5.8 (1.4)	51	6.6 (1.4)	0.002
Doi Y, Minowa M, Uchiyama M, et al. [30]	General Japanese adult male (20–29 years old)	103	PSQI score (mean, SD)	4.5 (2.1)	74	5.8 (3.1)	0.002
Tayama J, Nakaya N, Hamaguchi T, et al. [36]	General Japanese college students (male)	329	K6 score (mean, SD)	5.4 (4.5)	51	3.4 (4.1)	0.002
Asanuma T, Takeda F, Monma T, et al. [37]	Japanese college students in sports and physical education (male)	188	K6 score (mean, SD)	5.2 (4.6)	51	3.4 (4.1)	0.008
Umegaki Y, Todo N. [38]	General Japanese college students (male)	500	PHQ-9 score (mean, SD)	6.5 (5.2)	51	5.4 (4.6)	0.11
Japanese Ministry of Health, Labour and Welfare. [39]	General Japanese adult male (20–29 years old)	3869	K6 score => 5 (%)	24.7%	74	28.4%	0.46

Abbreviations: SD, standard deviation.

Welch's t-test (two-tailed test) was used to compare scores (PSQI, K6, and PHQ-9), and chi-square test was used to compare proportions.
